# Antibacterial Efficacy and Mechanisms of Curcumin-Based Photodynamic Treatment against *Staphylococcus aureus* and Its Application in Juices

**DOI:** 10.3390/molecules27207136

**Published:** 2022-10-21

**Authors:** Yuan Yuan, Qingyan Liu, Yanjun Huang, Mengyuan Qi, Haiyang Yan, Wenliang Li, Hong Zhuang

**Affiliations:** College of Food Science and Engineering, Jilin University, Changchun 130062, China

**Keywords:** aPDT, curcumin, inactivation kinetics, antibacterial mechanism, fruit juice

## Abstract

Antimicrobial Photodynamic Treatment (aPDT) is a non-thermal sterilization technology, which can inactivate common foodborne pathogens. In the present study, photodynamic inactivation on *Staphylococcus aureus* (*S. aureus*) with different concentrations of curcumin and light dose was evaluated and the mechanisms were also investigated. The results showed that curcumin-based aPDT could inactivate *S. aureus* cells by 6.9 log CFU/mL in phosphate buffered saline (PBS). Moreover, the modified Gompertz model presented a good fit at the inactivation data of *S. aureus*. Photodynamic treatment caused cell membrane damage as revealed by analyzing scanning electron microscopy (SEM) images. Leakage of intracellular constituents further indicated that cell membrane permeability was changed. Flow cytometry with double staining demonstrated that cell membrane integrity and the activity of nonspecific esterase were destroyed. Compared with the control group, intracellular reactive oxygen species (ROS) levels caused by photodynamic treatment significantly increased. Furthermore, curcumin-based aPDT reduced *S. aureus* by 5 log CFU/mL in juices. The color of the juices was also tested using a Chromatic meter, and it was found that b* values were the most markedly influenced by photodynamic treatment. Overall, curcumin-based aPDT had strong antibacterial activity against *S. aureus*. This approach has the potential to remove foodborne pathogens from liquid food.

## 1. Introduction

Microbial contamination is a big threat to food safety and public health. Outbreaks of food poisoning events due to microbes tend to increase in recent years. Among them, *Staphylococcus aureus* (*S. aureus*) accounts for 25% of the total, and it is the third largest microbial pathogen after *Haemophilus parahaemolyticus* and *Salmonella* [1]. As a common foodborne pathogen, *S. aureus* can grow in a wide range of temperatures and pH. When *S. aureus* reaches 5 log cfu/g in food, it may cause illness via toxin production. *S. aureus* may contaminate food even under extremely harsh conditions, leading to concerns among consumers [2]. Microbial contamination in foodstuffs has various sources, including the surface of processing equipment [3], food handlers’ behavior [4], water supply [5], and sanitation of food storage [6]. Thus, it is essential to establish an effective sterilization technology to minimize the risk of pathogenic bacteria.

Antimicrobial Photodynamic Treatment (aPDT) is an alternative sterilization technology, which has been widely used in medicine but its application in food is still limited [7]. Compared with conventional approaches, such as ultraviolet, pulsed electric fields, high-density carbon dioxide sterilization, aPDT shows a great potential for bacterial inactivation on foods [8,9], which will effectively reduce microbial threats, meeting consumers’ requirements for safe foods. Recently, more research has focused on the application of aPDT to inactivate pathogens, such as *Vibrio parahaemolyticus*, *Listeria monocytogenes,* and *Pseudomonas fluorescens* [10,11]. Furthermore, aPDT has been used to inactivate microorganisms on a series of fruit surface and meat to extend the shelf life [12,13]. However, on different food substrates, the effects of aPDT vary [14] and little is known about its effect on juices.

Photodynamic technology is based on the combined use of light, molecular oxygen, and vitally photosensitizer [13]. The mechanism is that the photosensitizer is activated with light of appropriate wavelength, in the presence of oxygen. After forming the excited singlet state, the activated photosensitizer will transit to the long-lived excited triplet state and undergo photochemical reactions [15]. During the aPDT process, a large number of cytotoxic reactive oxygen molecules (ROS), such as hydrogen peroxide, hydroxide radical, superoxide, and singlet oxygen [16], will be produced. ROS can react with DNA, proteins, lipids, and other components to produce cytotoxic effects and ultimately cause the death of organisms [17]. Since the attack occurs in a short time and numerous microbial targets that are simultaneously affected without toxic chemicals can be produced, it will not trigger bacterial resistance and has a broad spectrum of antibacterial effects [18,19]. In view of the complexity of the photodynamic process, the underlying mechanisms of aPDT inactivating foodborne pathogens have been explored, and most of these studies focus on changes in molecular mechanisms in organisms. Lai et al. [20] reported that photodynamic inactivation by curcumin-β-cyclodextrin complex involved bacterial DNA damage and protein degradation, and Kim and Yuk [21] suggested that ROS produced during aPDT damaged DNA by targeting guanine. Moreover, aPDT could significantly inhibit activity of intracellular oxidative defense enzymes [22].

Compared with the application in medicine, the food industry has higher requirements on the safety of photosensitizers. Among the common natural phenolic substances [23,24,25], curcumin is extracted from turmeric tubers and has a variety of biological activities, which has been approved as a food additive for flavoring and coloring. In addition, it has photosensitivity and can be activated by blue light to generate reactive oxygen species. Due to its natural origin and low cost, curcumin is considered to be a promising photosensitizer with broad application prospects. Therefore, our objective was to evaluate the antimicrobial efficacy of curcumin-based aPDT against *S. aureus* in phosphate buffered saline (PBS) and food models, and to elucidate the antimicrobial mechanisms from the perspective of cell morphology, cell membrane permeability change, intracellular ROS levels, and nonspecific esterase activity. We also attempted to determine the effect of curcumin-based aPDT on the colour of fresh juices. This study would help promote the application of photodynamic technology in food.

## 2. Results and Discussion

### 2.1. Antimicrobial Efficacy of aPDT in PBS against S. aureus

**Figure 1:**
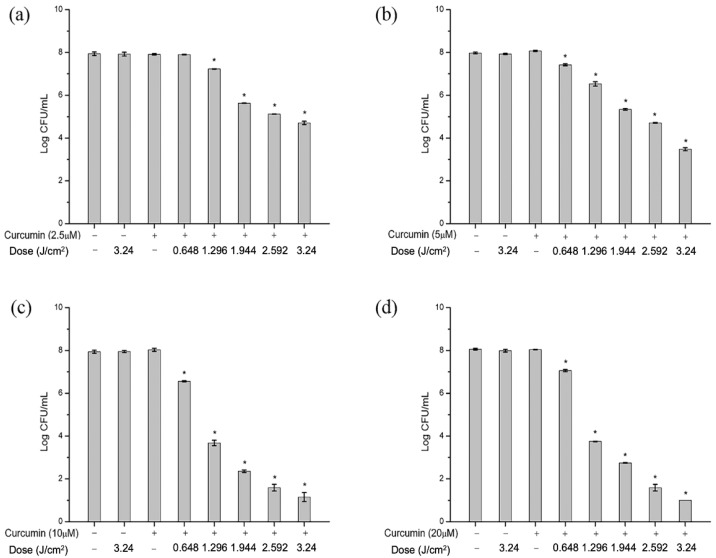
Effect of curcumin-based antimicrobial photodynamic treatment (aPDT) against *S. aureus* in PBS. (**a**–**d**) Different curcumin concentration (2.5, 5, 10 and 20 µM) under (0–3.24 J/cm^2^) illumination, respectively. Bars with an asterisk indicate significant difference (*p* < 0.05) compared to the negative control.

The antibacterial efficacy at different curcumin concentrations and light dose of aPDT against *S. aureus* in PBS was evaluated, as shown in Figure 1. The initial concentration of *S. aureus* was 10^8^ CFU/mL. Compared with the negative control, curcumin alone under the tested concentration did not significantly (*p* > 0.05) reduce the counts of *S. aureus*, and illumination treatment without a photosensitizer was similarly not effective. In contrast, after being exposed for 1.296 J/cm^2^, 2.5 µM curcumin-based aPDT significantly reduced *S. aureus* cells by 0.7 log CFU/mL of (Figure 1a, *p* < 0.05). With the increase of light dose, the antimicrobial effect was stronger, and the same trend was observed at other concentrations of curcumin (Figure 1b–d). The results suggested that light dose obviously influenced the curcumin-based antibacterial photodynamic effect. Figure 1 also demonstrates the strong impact of curcumin concentrations on the reduction in *S. aureus*. When the light dose was fixed at 1.944 J/cm^2^, with curcumin varying from 2.5 µM to 10 µM, *S. aureus* was reduced from 2.4 log CFU/mL to 5.3 log CFU/mL. Exceptionally, when the concentration was up to 20 µM, antimicrobial efficacy was not further increased. Increased concentrations saturated curcumin molecules, so the light penetration tended to decrease due to optical absorption [26,27]. The current result demonstrated the negative effects on aPDT antimicrobial efficacy at higher curcumin concentrations.

Obviously, the concentration of curcumin and light dose synergistically affected the antimicrobial efficacy of aPDT. Le and Nguyen [28] found that radiant fluence and the concentration of the photosensitizer affected the antibacterial effect, and they assessed the effect via calculating the number of absorbed photons. In addition, curcumin has a broad absorption spectrum in the range of 300–500 nm, with a maximum absorption wavelength of about 430 nm. Light sources in the blue region induce strong phototoxicity, which is the basis for the application of curcumin-mediated photodynamic technology in PBS [29,30]. Overall, the combination of 10 µM curcumin and 1.296 J/cm^2^ illumination treatment was the ideal aPDT condition for inactivating *S. aureus* in PBS. Photodynamic treatment was conducted under the abovementioned condition to investigate the mechanism of action, unless specially emphasized.

### 2.2. Inactivation Kinetics of aPDT in PBS against S. aureus

**Figure 2:**
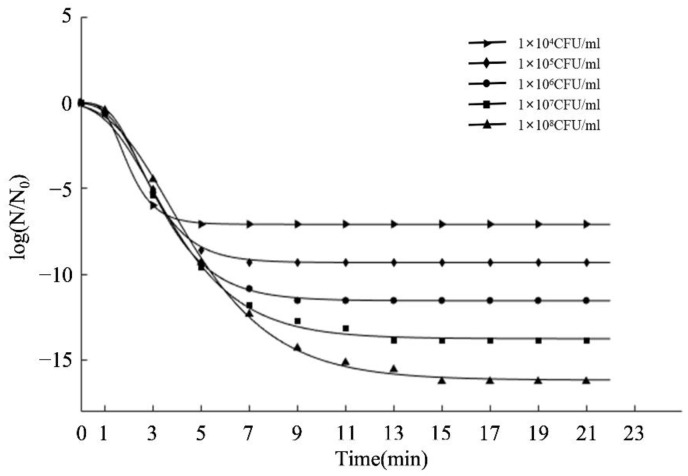
Effect of aPDT inactivating bacteria with 10 μM curcumin for 21 min in different initial concentration. N_0_: initial cell counts; N: viable cell counts after treatment.

To better understand the bactericidal characteristics, the reduction in viable cell count was fitted by the modified Gompertz model (Equation (1)). Inactivation kinetics of *S. aureus* with various inoculation concentrations after aPDT was generated from experimental data with log N/N_0_ (the logarithm ratio of viable cell count) versus time (illumination time), as shown in Figure 2. The biological parameters of A, k, K_dm_, and t_t_ related to the Gompertz equation, and R^2^ and RMSE were also obtained to analyze the goodness of fit (Table 1). It was obvious that the cell count of *S. aureus* in PBS decreased as the illumination time was extended to 21 min (Figure 2). It indicated that aPDT required a buffer period to take effect, followed by a rapid eradication of the bacteria until the end, when the cell count reached almost 1 log CFU/mL [31]. Additionally, λ, the lag phase time, was at around 1 min at all concentrations, which demonstrated that aPDT could inactivate *S. aureus* in a short time. For the curve of 1 × 10^4^ CFU/mL, only 3 min of illumination reduced *S. aureus* by 3.07 log CFU/mL and remained constant. However, the other initial bacterial densities required 7.8 min or less for complete inactivation (t_t_ value). Combining t_t_ and K_dm_ values (maximum inactivation rate), bacteria at lower concentrations were more vulnerable to photodynamic treatment (Table 1). Our previous study [32] about high voltage electrostatic field against *S. aureus* on medium plates found similar results, i.e., smaller initial bacterial density led to better antimicrobial effects. In addition, R^2^ and RMSE values suggested a good fit between the experimental data and the calculated values from the modified Gompertz equation.

### 2.3. Change of Cell Morphology of S. aureus after aPDT

**Figure 3:**
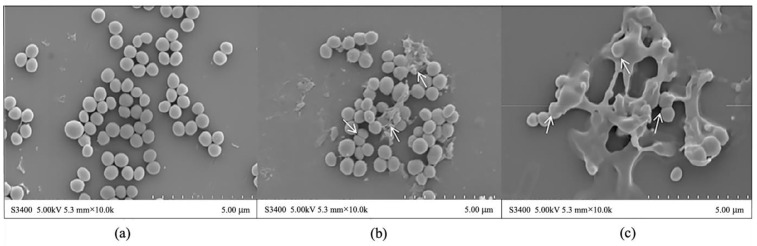
(**a**) SEM images of *S. aureus* untreated, (**b**) treated by aPDT for 1.296 J/cm^2^, and (**c**) treated by aPDT for 3.24 J/cm^2^.

*S. aureus* cells were analyzed by SEM to determine the effect of aPDT on the cell morphology (Figure 3). As revealed by Figure 3a, the surface of the untreated bacteria was smooth and took on a regular spherical shape. However, when the bacteria were illuminated for 1.296 J/cm^2^ dose, as shown by the arrow, the cell surface collapsed and some cellular components leaked out, accompanied by increased cell aggregation (Figure 3b). Similar results were found by Buchovec et al. [33] that cells shrank after aPDT, and their results pointed that the leaked material was likely to be intracellular DNA or protein drained out of the cell following membrane damage. As the light dose increased to 3.24 J/cm^2^, damage on the surface of *S. aureus* obviously strengthened, and cells were unidentifiable or killed (Figure 3c). Cabiscol et al. [34] reported that ROS produced by aPDT could attack the polyunsaturated fatty acids of bacterial membranes and cause lower membrane fluidity, which eventually led to membrane degradation, in agreement with our results. The SEM images reflect that photodynamic treatment led to cell rupture and affected the permeability of the cell membrane, which lays a foundation for the subsequent study of photodynamic mechanism.

### 2.4. Effect of aPDT on Membrane Permeability of S. aureus

**Figure 4:**
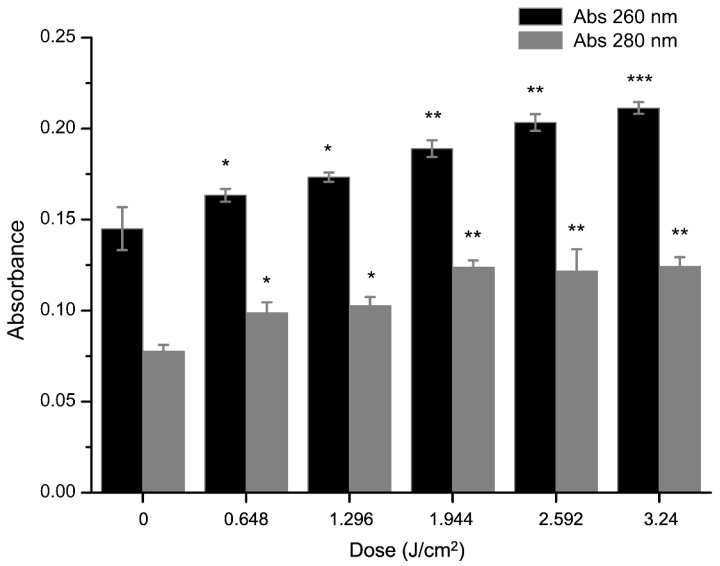
Effect of aPDT for different light dose (0, 0.648, 1.296, 1.944, 2.592, 3.24 J/cm^2^) on the leakage of DNA and protein. Bars with an asterisk indicate significant difference (*p* < 0.05) compared to the control group. The more asterisks, the stronger the significance.

A classic indication of bacterial cytoplasmic membrane damage is the leakage of cytoplasmic contents [35]. The effects of curcumin-based aPDT on the leakage of DNA and protein were measured by OD_260_ and OD_280_. As shown in Figure 4, the supernatant was significantly (*p* < 0.05) changed, compared with the control group. After illumination for 1.296 J/cm^2^, the OD values of DNA and protein in the bacterial supernatant increased by 0.044 and 0.046, respectively. Similarly, the OD values increased with dose, indicating a continuous increase in DNA and protein leakage, and the maximum value was at 3.24 J/cm^2^ (Figure 4). A previous study [36] had the same results. With the extension of illumination time, the leakage of cytoplasmic contents induced by aPDT was markedly increased, and adding extra EDTA would enhance this effect. Taken together, we conclude that these spectral changes might result from the damage of cell membrane.

### 2.5. Effect of aPDT on Membrane Integrity and Esterase Activity of S. aureus

**Figure 5:**
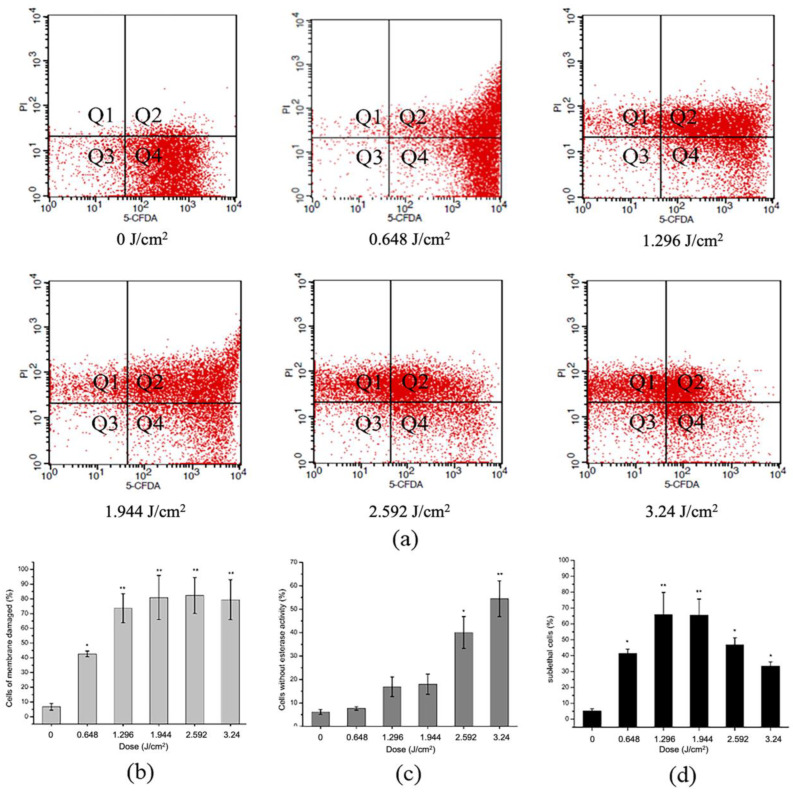
(**a**) Effect of aPDT against *S. aureus* with different light dose (0, 0.648, 1.296, 1.944, 2.592, 3.24 J/cm^2^), which membrane integrity and esterase activity indicated by flow cytometry with cFDA and PI; (**b**) showed the proportion of membrane damaged cells; (**c**) showed esterase activity lost cells; and (**d**) sublethal cells in the flow cytometry investigation. Bars with an asterisk indicate significant difference (*p* < 0.05) compared to the control group. The more asterisks, the stronger the significance.

Two fluorescent probes PI and cFDA were selected to investigate the effect of aPDT on the cell membrane structure of *S. aureus* (Figure 5). PI stains DNA or RNA inside dead cells or ones with damaged membranes, cFDA stains live cells with esterase activity, while sublethally injured cells with compromised membranes but still with esterase activity can be stained by both probes. Changes in cell number in different fluorescent regions with increasing light dose reveal an altered cell survival status [37,38].

As shown in Figure 5, the untreated cells which were only stained by cFDA appeared in large numbers in Q4, indicating that the esterase enzyme was active and membranes were intact (Figure 5a). More cells appeared in Q1 and Q2 after aPDT, suggesting that membrane permeabilization was impaired, and PI freely entered the cell. The proportion of damaged membranes obviously enhanced with light dose (Figure 5b). In addition, massive cells shifted to Q1 and Q3, where the cells lost esterase activity (Figure 5c). During aPDT, the amount of sublethal cells in Q2 rose up first and declined later, reaching the maximum at 1.296 and 1.944 J/cm^2^ dose (Figure 5d). We speculated that it was due to the accumulation of photodynamic effects, which caused cells to shift from the sub-injury state to death. These sub-injury cells are often missed in routine testing, but can resume growth and multiply when conditions are suitable, posing a safety risk. Thomas et al. [39] achieved similar results to ours, concluding that the plasma membrane is a prime target for antimicrobial aPDT and that initial membrane photodamage will facilitate both photosensitizers influx and leakage of metabolites out of the cell until a sufficient treatment time to completely kill the bacteria. Overall, as evidenced in these data, aPDT showed illumination-dependent disruptive effects on membrane integrity and esterase activity, which eventually led to cell death.

### 2.6. Effect of aPDT on Reactive Oxygen Species (ROS) of S. aureus

It is well-recognized that ROS can damage cellular structures, inhibit the function of subcellular structures, and ultimately result in cell death [40]. To further sort out the mechanisms of curcumin-based aPDT inactivation of *S. aureus*, intracellular change of ROS was detected by specific probe DCFH. The nonfluorescent DCFH-DA can permeate into cells and ultimately be oxidized to fluorescent 2′,7′-dichlorofluorescin (DCFH) by ROS [41].

**Figure 6:**
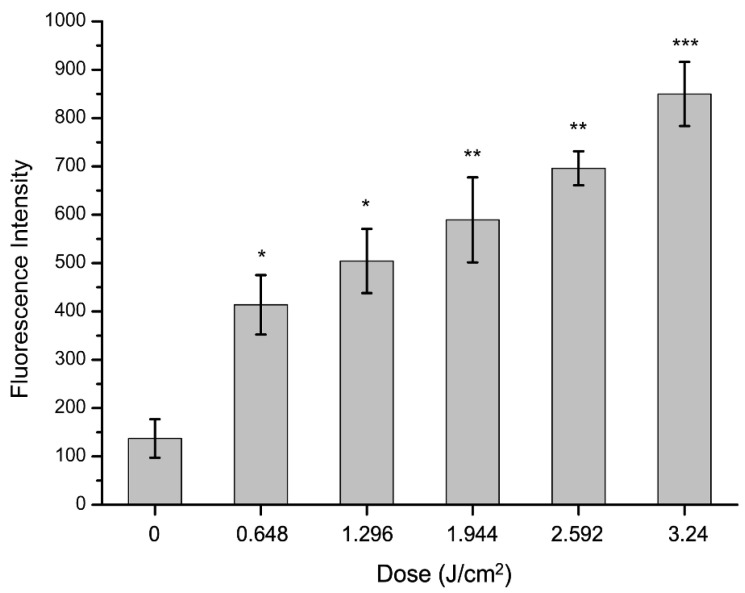
Intracellular ROS of *S. aureus* after aPDT for different light dose (0, 0.648, 1.296, 1.944, 2.592, 3.24 J/cm^2^). Bars with asterisks indicate significant difference (*p* < 0.05) compared to the control group. The more asterisks, the stronger the significance.

*S. aureus* suspension was treated as described above. After illumination for different dose with 10 µM curcumin, the ROS level was measured. Compared with the untreated sample, fluorescence led to a significant rise (*p* < 0.05) with the increase of light dose (Figure 6). Thus, photodynamic treatment resulted in an increase of intracellular ROS content. Of note, as impacted by aPDT, intracellular ROS levels nearly altered in a consistent fashion with bacterial mortality, implying a causal-effect relevance. Research discovered that the effect of aPDT was significantly compromised when ROS scavengers were added, indicating that ROS were critical for killing bacteria [40]. In conclusion, the accumulation of intracellular ROS damaged the functionality of the cell membrane and caused its disruption, as a consequence of cell death. 

### 2.7. Antibacterial Efficacy of aPDT in Juices against S. aureus

**Figure 7:**
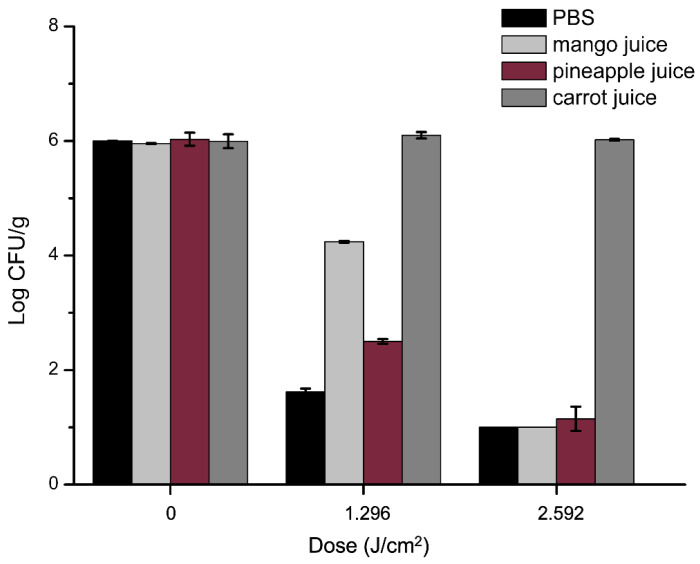
Effect of the curcumin-based aPDT against *S. aureus* in mango, pineapple, and carrot juice at 10 µM curcumin with 1.296 and 2.592 J/cm^2^ light dose. Samples of PBS were used as control.

The experiments were conducted as previously described for PBS experiments with some modifications. As shown in Figure 7, antibacterial efficacy of aPDT in three kinds of juice was tested, and aPDT in PBS was also implemented as control. The curcumin alone did not exhibit any antibacterial activity against *S. aureus*, while after 1.296 J/cm^2^ dose of aPDT, *S. aureus* decreased by 1.8 and 3.5 Log CFU/mL in mango and pineapple juice, respectively. As the light dose increased to 2.592 J/cm^2^, the significant (*p* < 0.05) change was that neither mango nor pineapple juice showed detected cells. These were consistent with our previous results in PBS, i.e., light dose significantly influenced bactericidal effects. However, the antimicrobial efficacy in juice was lower than that in PBS, as visualized in Figure 7. It is owing to the turbidity of the juice compared to PBS, which impacted light penetration in the process of aPDT [42,43]. The low transmission depth of the blue light source is a limiting factor for the efficiency of aPDT in juices [44]. The absorption and reflection of the light source by colored compounds and soluble solids in liquid food will decrease the transmission depth of the light source and the antibacterial effect [45]. Previous studies had similar reports that apple juice was more turbid than tested grape juice, which resulted in an overall lower number of decimal reductions of spores [46]. Moreover, aPDT was more efficient in pineapple than in mango juice. More interestingly, 12 min with 10 µM curcumin did not have any effect on carrot juice. It was due to the presence of organic compounds in juice, which can quench ROS, interfered with the photodynamic reactions [22]. In particular, carotenoids, which are abundant in carrot juice, dramatically reduce the sensitivity of *S. aureus* in carrot juice to aPDT by its efficient antioxidant activity [44,47]. The disinfection effect of aPDT has also been found in other fruits. Lin et al. [48] and Sheng et al. [49] found that aPDT could reduce the microbial count on fresh-cut Hami melons and lemon surfaces. It is noteworthy that aPDT mainly works on microorganisms in fruit juices or on fruit surfaces. It can be inferred that photodynamic technology provides an alternative to the inactivation of pathogenic microorganisms in food, and the germicidal efficacy in juices not only depends on the concentration of photosensitizer and light dose but also the characteristics of food itself, such as turbidity, color, etc., and the factors need to be further explored.

### 2.8. Effects of the aPDT on the Color Changes of Juice

Color is a crucial factor determining food market value, and also serves as an indicator of food quality during processing and storage of a fruit juice [50]. The changes of total color difference (Δ*E*) of the photodynamic juices are shown in Table 2. During aPDT, the main change occurred to a* values, suggesting that yellowness was the main parameter influenced by photodynamic, and resulted in a masking effect on Δ*E*, and L* and a* values were less changed. As a result, the yellow pigment in juice was degraded under the influence of light [51]. However, decrease in the yellow value made the color of the juice more natural, which could be a benefit in terms of visual preference. b* values of juice decreased during the photodynamic process, illustrating there was a certain photobleaching in the application of aPDT in juice, which has an influence on juice quality. The color change was also observed in other studies on fruit surfaces and juice after LED illumination [52,53]. Fortunately, the decline rate tended to slow down with time, and this color change in food could be reduced by optimizing the selection of irradiance and temperature [54]. In addition, carrot juice after treatment slightly changed in color, which could be ignored. Combined with our previous results, we speculate color changes could be associated with the antimicrobial effect.

## 3. Materials and Methods

### 3.1. Bacterial Strains and Culture Conditions

*S. aureus* ATCC 29213 was obtained from China Medical Culture Collection Center (Beijing, China) and stored at −20 °C. Frozen cultures were activated by inoculating with a loopful of inoculum onto a Tryptic soy agar (TSA) medium and incubated at 37 °C for 24 h. A single colony was then enriched in 20 mL sterile Tryptic soy broth (TSB) and grown overnight at 200 rpm on an orbital shaker (HY-5, JinBo Equipment Industry Co., Jiangsu, China) at 37 °C for 12 h. Then, the culture was centrifuged at 6000× *g* for 5 min in a centrifuge (JW-3021HR, Anhui Jiaven Equipment Industry Co., Anqing, China) at 4 °C. Finally, the resultant pellets were washed twice and redissolved with sterilized phosphate buffered saline (PBS, Meilun Biotechnology, Dalian, China) to obtain a suspension of about 6 × 10^9^ CFU/mL for later use.

### 3.2. Photosensitizer and Light Sources

Curcumin with purity of ≥97% (CAS 458-37-7, Meilun Biotechnology Co. Ltd., Dalian, China) was dissolved in DMSO (Sangon Biotech Co., Ltd., Shanghai, China) to obtain a stock solution of 10 mM. The stock solution was maintained in the dark at −20 °C and used within two weeks. Before each experiment, curcumin solutions were diluted with PBS and stirred for 5 min on a magnetic stirrer to different concentrations of 2.5, 5, 10 and 20 µM.

Blue light-emitting diode arrays (440 ± 5 nm, JiaDeng Lighting, Hangzhou, China) were used as the light source for aPDT. In the experiment, 15 mL of the sample solution was deposited in a sterile petri dish (90 mm in diameter) and placed on a platform below the light source. The power density received by samples was 3.6 × 10^−3^ W/cm^2^, which was measured with a hand-held solar power meter (SM206-SOLAR; Xinbao Technology, Shenzhen, China). The light dose (in J/cm^2^) was calculated by multiplying the power density (in W/cm^2^) by illumination time (in seconds).

### 3.3. Antibacterial Efficacy of aPDT in PBS

Photoinactivation experiments were performed according to Prasad et al. [55] with some modifications. *S. aureus* suspension was adjusted to about 10^8^ CFU/mL and mixed with curcumin solution (2.5, 5, 10, 20 μM) for 2–3 min, then incubated in the dark for 30 min and exposed to light for aPDT. Photodynamic treatment involved two parameters’ variations, the concentration of curcumin with the wide range from 2.5 to 20 μM, and the light dose from 0 to 3.24 J/cm^2^. All aPDT experiments were compared with three independent control groups: negative control (light−, Cur−), Cur control (light−, Cur+) and light control (light+, Cur−). After aPDT, the suspension was 10-fold serially diluted in sterile PBS and spread evenly by a disposable L-type spreading rod (BIOLOGIX, Kansas, KS, USA) on Baird-Parker agar (BP) plates and incubated at 37 °C for 24 h. The surviving cells were enumerated and reported by Log CFU/mL. Each sample was tested at least in triplicate.

### 3.4. Inactivation Kinetics of aPDT

*S. aureus* suspension was diluted in PBS and adjusted to 1 × 10^8^, 1 × 10^7^, 1 × 10^6^, 1 × 10^5^, and 1 × 10^4^ CFU/mL, respectively. Bacterial suspension at each concentration was mixed with 10 µM curcumin, then incubated in the dark for 30 min and exposed to light under a series of time gradients (0, 1, 3, 5, 7, 9, 11, 13, 15, 17, 19, and 21 min, respectively) for aPDT. After aPDT, the suspension was 10-fold serially diluted in sterile PBS and incubated on BP plates at 37 °C for 24 h. The cells were enumerated by CFU/mL. Inactivation kinetics of aPDT at different bacterial inoculation concentrations was obtained by employing the modified Gompertz equation [31], which is described as follows:

(1) $$\log N/N_{0}\;=\;A\;\times \;exp\left[-exp\right]$$

where log *N*/*N*_0_ is the natural logarithmic ratio of viable cell count. A (−*log N_max_*) is the lower asymptote value. The time required for complete inactivation is defined by t_t_. At the time of complete inactivation, log *N*/*N*_0_ = A, with the tangent through the inflection point. Thus, t_t_ can be calculated as:

(2) $$t_{t}\;=\;\lambda \;+\;A/K_{dm}$$

where *K_dm_* is the maximum inactivation rate (min^−1^), k is the time for the lag phase, and t is the treatment time.

### 3.5. Morphology Alteration of S. aureus Observed by Scanning Electron Microscopy (SEM)

To assess the alteration of *S. aureus* membrane morphology, scanning electron microscopy was performed based on the method reported by Jan et al. [56] with some modifications. *S. aureus* of 10^8^ CFU/mL was incubated with 10 µM of curcumin and then incubated in the dark for 30 min. Then, 1.296 and 3.24 J/cm^2^ of light dose were selected as described above. After aPDT, the untreated and treated cells were transferred into a 24-well polystyrene microplate (Corning, NY, USA) containing glass coverslips and incubated at 37 °C for 1 h. The coverslips were gently washed with PBS twice and fixed in glutaraldehyde (2.5% in 0.01 M phosphate buffer, pH 7.2) for 12 h at 4 °C. Next, the coverslips were dehydrated in ethanol in a gradient (30, 50,70, 80, 90, 95, 100%, *v*/*v*) and rinsed with tertiary butanol for 20 min. The resulting samples were freeze-dried, sputter-coated with gold, and observed using a SEM (FESEM, S-3400 N, Hitachi, Japan).

### 3.6. Measurement of Intracellular Protein and DNA Leakage

The leakage of intracellular protein and DNA was measured according to the method of Hu et al. [36] with some modifications. *S. aureus* suspension was adjusted to about 10^8^ CFU/mL and mixed with 10 µM curcumin solution, and then incubated in the dark for 30 min. After aPDT for 3.24 J/cm^2^ dose, culture from treated or control groups was centrifuged at 5000× *g* for 5 min immediately. The OD value of collected supernatant was measured using a spectrophotometer (Bio-Rad Laboratories, Hercules, CA, USA) at wavelength of 260 nm and 280 nm, respectively.

### 3.7. Flow Cytometry for Observation of S. aureus Membrane Integrity and Esterase Activity

Flow cytometry was performed according to the methods of Li et al. [38] with some modifications. Mixture of 10^8^ CFU/mL *S. aureus* suspension and 10 µM curcumin solution was incubated in the dark for 30 min and exposed to aPDT as described above. Subsequently, the cultures untreated and treated were dyed with 100 µM 5-Carboxyfluorescein diacetate (5-CFDA, Thermo Fisher Scientific, Waltham ME, USA) and 15 µM propidium iodide (PI, Thermo Fisher Scientific, Waltham ME, USA) in the dark at 37 °C for 15 min. After washing with PBS, the samples were run on flow cytometer (BD FACSCalibur, America). The flow cytometer excitation wavelength was 488 nm, detector FL1 detected at 525 nm, and detector FL3 detected at 650 nm. In total, 10,000 cells were collected for each sample, and the fluorescence signals of 5-CFDA and PI were detected in the FL1 and FL3 channels, respectively. The data were analyzed by BD CellQuest Pro software and represented as bivariate dot plots.

### 3.8. Change of Reactive Oxygen Species (ROS) of S. aureus after aPDT

*S. aureus* cells were treated with aPDT and processed as described in flow cytometry experiments. The change of ROS intracellular *S. aureus* was determined using 2′,7′-dichlorofluorescin-diacetate (DCFH-DA, purity ≥97%, Sigma-Aldrich, St Louis, MO, USA) as ROS probe, which was immediately added to cultures to a final concentration of 5 µM. The mixture was incubated at 37 °C in the dark for 30 min [57]. ROS fluorescence was measured using a fluorescence spectrophotometer (Hitachi F-3000, Japan), whose excitation wavelength was at 485 nm and emission wavelength was at 528 nm. Fluorescence values of stained bacterial suspensions were corrected by subtracting the means of fluorescence of stained PBS.

### 3.9. Antibacterial Efficacy of aPDT in Juices

Freshly squeezed juices (mango, pineapple, and carrot juice) in the absence of preservation agent were purchased from a local supermarket in Changchun, Jilin and used within one day. *S. aureus* suspension was centrifuged at 6000× *g* for 5 min at 4 °C and resuspended in juice to a final concentration of 1 × 10^6^ Log CFU/mL. The same treatment was performed with sterile PBS as a control. The bacterial suspension was mixed with 10 µM curcumin solution, then incubated in the dark for 30 min and exposed for 1.296 and 2.692 J/cm^2^ light dose. Meanwhile, samples without illumination were also placed in an incubator. At the end of the experiment, 0.1 mL of treated juice or PBS was spread evenly on BP plate after diluting serially. Plates were incubated at 37 °C for 24 h, and then colonies were enumerated and reported as log CFU/mL.

### 3.10. Color Analysis of Juice after aPDT

Color change of the juice was monitored using a Chromatic meter SC-80C (Kangguang Instrument Co., Ltd., Beijing, China) according to Fundo et al. [58]. The equipment was calibrated before every experiment with a blank calibration tile, and the samples were exposed for aPDT as described above. All experiments were conducted at least in triplicate. The samples were treated in the dark to avoid quality changes caused by light. The color was represented by L*, a*, and b* parameters, which separately represented luminosity, chromaticity on the green (−) to red (+) axis, and chromaticity on the blue (−) to yellow (+) axis. The whiteness of the juice was evaluated by b*. The difference in total color difference (△*E*) was obtained according to the following equation:

(3) $$△E\;=\;{\left[{\left(L^{*}\;-\;L_{0}\right)}^{2}\;+\;{\left(a^{*}\;-\;a_{0}\right)}^{2}\;+\;{\left(b^{*}\;-\;b_{0}\right)}^{2}\right]}^{1/2}$$

In Equation (3), the number “0” is the initial color values from untreated juice samples.

### 3.11. Statistic Analysis

All experiments were carried out in independent triplicate with duplicate sampling (*n* = 6). Statistical analysis was performed using SPSS 25 software (SPSS Inc., Chicago, IL, USA). Data were expressed by mean ± standard deviation. Significant differences in the mean values were calculated at the 95% confidence interval (*p* < 0.05) using one-way analysis of variance (ANOVA), and means were separated by least significant difference (LSD). Graphs were drawn with Origin Pro 8.0 software (Origin Lab Corporation, Northampton, MA, USA).

## 4. Conclusions

In summary, curcumin-based aPDT effectively inactivated *S. aureus* in PBS and juice, whereas the effect was diminished in the food matrix. In addition, it destroyed cell morphology and esterase activity, leading to leakage of cellular contents and increasing the intracellular ROS content, but the localization of the photosensitizer curcumin on bacteria and the specific pathway of aPDT-induced oxidative cell damage remain to be investigated, which is the direction of our upcoming research. Photodynamic technology provides a potential for killing food-borne pathogens in liquid foods; however, the efficacy of aPDT in food matrix needs to be enhanced, and synergies with other thermal or non-thermal technologies are expected. This study would help promote the application of photodynamics in the food industry.

## Figures and Tables

**Table 1:** Estimation of the kinetic parameters of the modified Gompertz equation for the inactivation of *S. aureus* in PBS after aPDT.

Initial Density (CFU/mL)	A	K_dm_ (min^−1^)	λ (min)	t_t_ (min)	R^2^	RMSE
1 × 10^8^	16.15	2.422	1.217	7.885	0.9987	0.2467
1 × 10^7^	13.74	2.471	0.927	6.487	0.9977	0.2529
1 × 10^6^	11.53	2.821	1.158	5.245	0.9998	0.063
1 × 10^5^	9.308	2.948	1.212	4.369	0.999	0.1114
1 × 10^4^	7.082	3.493	0.924	2.952	0.9999	0.0256

**Table 2:** Estimated color parameters for untreated and light-exposed juice (mango, pineapple, and carrot juice) for 1.296 and 2.592 J/cm^2^ light dose, respectively.

Sample	Treatment	L*	a*	b*	Δ*E*
Mango juice	control	58.9 ± 0.01 ^b^	3.59 ± 0.021 ^b^	42.17 ± 0.14 ^a^	
	1.296 J/cm^2^	59.4 ± 0.059 ^a^	3.83 ± 0.075 ^a^	35.18 ± 0.046 ^b^	7.01 ± 0.112
	2.592 J/cm^2^	59.3 ± 0.023 ^a^	3.69 ± 0.012 ^a^	31.86 ± 0.058 ^b^	10.32 ± 0.085
Pineapple juice	control	79.1 ± 0.064 ^b^	−1.72 ± 0.025 ^b^	30.99 ± 0.235 ^a^	
	1.296 J/cm^2^	80.1 ± 0.071 ^a^	−0.60 ± 0.025 ^a^	22.40 ± 0.214 ^b^	8.72 ± 0.0601
	2.592 J/cm^2^	79.3 ± 0.127 ^a^	−0.02 ± 0.046 ^a^	18.56 ± 0.125 ^b^	12.54 ± 0.139
Carrot juice	control	16.1 ± 0.001	22.7 ± 0.001	20.3 ± 0.001	
	1.296 J/cm^2^	16.1 ± 0.006	22.7 ± 0.025	20.6 ± 0.315	0.34 ± 0.316
	2.592 J/cm^2^	16.1 ± 0.001	22.7 ± 0.018	20.4 ± 0.116	0.07 ± 0.11

The values are average ± SD. For a given parameter, values with different letters different significantly (*p* < 0.05). ΔE, total color change.

## Data Availability

Not applicable.

## Sample Availability

Samples of the compounds are not available from the authors.
